# NF135.C10: A New *Plasmodium falciparum* Clone for Controlled Human Malaria Infections

**DOI:** 10.1093/infdis/jis725

**Published:** 2012-11-27

**Authors:** Anne C. Teirlinck, Meta Roestenberg, Marga van de Vegte-Bolmer, Anja Scholzen, Moniek J. L. Heinrichs, Rianne Siebelink-Stoter, Wouter Graumans, Geert-Jan van Gemert, Karina Teelen, Martijn W. Vos, Krystelle Nganou-Makamdop, Steffen Borrmann, Yolanda P. A. Rozier, Marianne A. A. Erkens, Adrian J. F. Luty, Cornelus C. Hermsen, B. Kim Lee Sim, Lisette van Lieshout, Stephen L. Hoffman, Leo G. Visser, Robert W. Sauerwein

**Affiliations:** 1 Department of Medical Microbiology, Radboud University Nijmegen Medical Center, and Departments of; 2 Medical Microbiology (Clinical Microbiology Laboratory); 3 Parasitology; 4 Infectious Diseases, Leiden University Medical Center, the Netherlands; 5 Department of Infectious Diseases, Heidelberg University School of Medicine, Germany; 6 Sanaria Inc, Rockville, Maryland

**Keywords:** Malaria, parasite culture, parasite strain, parasite clone, field strain, cellular immunology, clinical trial, controlled human malaria infection

## Abstract

We established a new field clone of *Plasmodium falciparum* for use in controlled human malaria infections and vaccine studies to complement the current small portfolio of *P. falciparum* strains, primarily based on NF54. The Cambodian clone NF135.C10 consistently produced gametocytes and generated substantial numbers of sporozoites in *Anopheles* mosquitoes and diverged from NF54 parasites by genetic markers. In a controlled human malaria infection trial, 3 of 5 volunteers challenged by mosquitoes infected with NF135.C10 and 4 of 5 challenged with NF54 developed parasitemia as detected with microscopy. The 2 strains induced similar clinical signs and symptoms as well as cellular immunological responses.

***Clinical Trials Registration*** NCT01002833.

Malaria caused an estimated 216 million cases and approximately 1 million deaths in 2010 [1], mainly in sub-Saharan Africa where most cases are caused by *Plasmodium falciparum*. Development of vaccines and new drugs and better understanding of immunological processes are essential to tackling this immense problem. Controlled human malaria infection (CHMI), in which healthy volunteers are exposed to bites of *P. falciparum–*infected mosquitoes, is a powerful tool to address questions regarding *P. falciparum* drug and vaccine efficacy*,* clinical signs and symptoms, parasite kinetics, and human immunology. Since the first CHMI by mosquitoes fed on cultures of *P. falciparum*, >1300 healthy volunteers have been exposed to CHMI with mainly the Nijmegen falciparum strain NF54 or its clone 3D7 [2]. Strain/parasite line NF54 stably produces sexual stages required for production of infectious mosquitoes. Parasites have been adapted to laboratory conditions by continuous in vitro culture for >3 decades. In the field, *P. falciparum* displays a wide genetic diversity, which is currently not represented by the available laboratory strains for CHMI. Other strains, including the South American 7G8 *P. falciparum* clone of the Brazilian strain IMTM22, have been sporadically used in limited number of volunteers [3–5]. We therefore aimed to identify, clone, and test an additional *P. falciparum* strain that can be used in CHMIs, and we developed several qualification criteria: The clone *(a)* must consistently produce gametocytes and sporozoites, *(b)* should be cloned to create a single genetically homogenous parasite population, *(c)* should be sensitive to commonly administered antimalarials, and *(d)* should be of non-African origin to be geographically and genetically distinct from the NF54 strain, an airport strain that probably originates from Africa [6]. Here we report the generation, characterization, and first CHMI for NF135.C10, a new Cambodian clone; findings include drug sensitivity, microsatellite profile, kinetics of parasitemia, and clinical and immunological properties in a direct comparison with NF54.

## METHODS

Blood collected from patients for diagnosis of malaria was cultured in Roswell Park Memorial Institute 1640 medium containing 10% human serum at 5% hematocrit in a semiautomated suspension culture system, cloned by limiting dilution, and fed to *Anopheles stephensi* mosquitoes, reared according to standard operating procedures, as described elsewhere [7]. Salivary glands of 10 mosquitoes were dissected for each strain to confirm the presence of sporozoites. The identities of NF135.C10 and NF54 were defined by polymerase chain reaction (PCR), microsatellite mapping of the *P. falciparum* rifin repetitive microsatellite (pfRRM), and drug sensitivity assay. (For descriptions of the respective techniques, please refer to the Supplementary Methods.)

Volunteers, aged 18–35 years, were screened at the Leiden University Medical Centre for eligibility based on medical and family history, physical examination, and general hematological and biochemical tests. All volunteers gave written informed consent before inclusion.

Ten Dutch malaria-naive volunteers were randomized into 2 groups and exposed to bites of 5 *A. stephensi* mosquitoes infected with either NF54 or NF135.C10 for 10 minutes. Feeding sessions were repeated until each volunteer had been bitten by exactly 5 *P. falciparum–*infected mosquitoes.

Starting on day 5 after infection, volunteers were subjected to intensive outpatient follow-up with up to thrice-daily visits. Signs and symptoms (solicited and unsolicited) were recorded and graded by the attending physician as follows: mild (easily tolerated), moderate (interferes with normal activity), or severe (prevents normal activity) or, for fever, grade 1 (>37.5°C to 38.0°C), 2 (>38.0°C to 39.0°C), or 3 (>39.0°C). Hematological and biochemical parameters were monitored daily. After identification of a positive blood smear, or if smears remained negative until day 21, volunteers were treated with a curative regimen of atovaquone and proguanil (1000 and 400 mg/d, respectively) for 3 days. The trial was performed in accordance with good clinical practice and approved by the Central Committee for Research Involving Human Subjects of The Netherlands (CCMO NL30350.058.09).

Thick blood smears were examined by microscopy twice daily on days 5 and 6 after challenge, thrice daily on days 7–11, twice daily on days 12–15, and once daily on days 16–21. For each smear, 15 µL of ethylenediaminetetraacetic acid–anticoagulated blood was stained with Giemsa for 30 minutes and examined at ×1000 magnification, with assessment of approximately 0.5 µL of blood. A smear was considered positive if 2 unambiguously identifiable parasites were found. The prepatent period was defined as the period between exposure to infected mosquitoes and the first positive blood smear. Parasitemia was also measured retrospectively with real-time quantitative PCR (qPCR), using a technique described elsewhere [8], with minor changes (the MGB probe AAC AAT TGG AGG GCA AG was used instead of the turbo TaqMan probe sequence).

In vitro immunological assays were performed on peripheral blood mononuclear cells isolated from venous whole blood on the day before challenge, on days 5, 35, and 140 after challenge, and on the first day of treatment. Cells were stored in liquid nitrogen and, after thawing, cultured in the presence of NF135.C10 or NF54 *P. falciparum* red blood cells (RBCs) at a 1:2 ratio (peripheral blood mononuclear cells to *P. falciparum* RBCs) for 24 hours. Flow cytometric staining was performed for CD4, CD45RO, CD3, CD62L, CD8a, γδ T-cell receptor, CD56, interferon γ (IFN-γ), tumor necrosis factor, and interleukin 2. A more detailed description can be found in the Supplementary Methods.

Data were analyzed using GraphPad Prism5 software (GraphPad). Differences in parasite kinetics between subjects in the NF135.C10 and NF54 groups were analyzed using the nonparametric Mann–Whitney *U* test. Differences were considered statistically significant at *P* < .05 (2 sided).

## RESULTS

*Plasmodium falciparum* strains obtained from 74 patients with malaria were adapted to culture; 21 strains produced gametocytes, and 16 were able to infect mosquitoes. Based on gametocyte production, exflagellation, and transmission to mosquitoes, 7 strains were cloned. Two of these clones produced at least 5 oocysts and 30 000 sporozoites in >70% of mosquitoes. The drug sensitivity profile of NF135.C10 is similar to that of NF54 for atovaquone, proguanil, dihydroartemisinin, and lumefantrine, but NF135.C10 is >8-fold less sensitive to chloroquine than NF54. The culture characteristics and drug sensitivity of NF135.C10 and NF54 are shown in Table 1. Comparison of NF135.C10 and NF54 genotypes using PCR and rifin microsatellite mapping showed distinct genetic profiles (Supplementary Figure 1).

**Table 1.** NF135.C10 and NF54 Culture Characteristics

	NF135.C10	NF54
Restarted cultures until CHMI	7	306
Country of origin	Cambodia	West Africa (airport)
Year of isolation	1993	1979
Period 2009–2010^a^		
Infection, %	74 (62–87)	86 (78–94)
Oocysts	12 (7.3–16)	27 (22–33)
Sporozoites/mosquito, ×10^3^	39 (18–60)	99 (74–124)
CHMI (April 2010)		
Infection, %	100	100
Oocysts	5.6	17
Sporozoites/mosquito, ×10^3^	12.5	69
Gametocyte male-female ratio	1:5	1:3
Drug sensitivity, mean IC_50_ (SD)^b^		
Dihydroartemisinin, nmol/L	3.4 (1.8)	9.9 (6.0)
Lumefantrine, nmol/L	89 (26)	78 (9.7)
Proguanil, µmol/L	21 (3.3)	27 (4.0)
Atovaquone, nmol/L	0.3 (0.1)	0.6 (0.3)
Chloroquine, nmol/L	201 (45)	24 (1.1)

Abbreviation: CHMI, controlled human malaria infection; IC_50_, half-inhibitory concentration; SD, standard deviation.

Mosquito infection and drug sensitivity profiles of NF135.C10 and NF54 in the period 2009–2010 and for the specific batches used in this CHMI.

^a^ Data for the period 2009–2010 represent mean findings (95% confidence intervals) after 26 and 39 standard dissections, for NF135.C10 and NF54 respectively, from 10 mosquitoes per dissection.

^b^ The drug sensitivities of NF135.C10 and NF54 were tested by the malaria SYBR Green I–based fluorescence assay in triplicate experiments; values represent means from 3 independent experiments.

**Figure 1.**
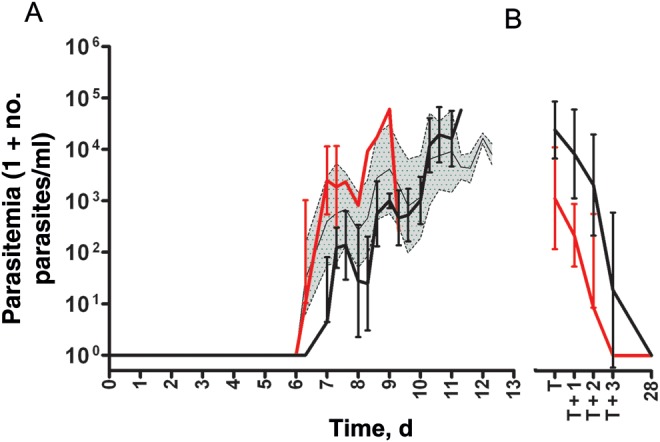
Parasite kinetics of *Plasmodium falciparum* strains NF135.C10, and NF54 assessed by quantitative real-time polymerase chain reaction. Volunteers were infected by bites of mosquitoes infected with either NF135.C10 or NF54. *A,* Parasitemia of volunteers until thick smear positivity; data are shown as geometric means and 95% confidence intervals for volunteers successfully infected with NF135.C10 (*red*) or NF54 (*black*) and historical controls infected with NF54 (*gray area*; n = 48). *B,* Parasitemia of volunteers at the time of smear positivity and subsequent start of treatment (T), followed up for 3 days and finally at 28 days after infection; data are shown as geometric means and 95% confidence intervals for volunteers successfully infected with NF135.C10 (*red*; n = 3) or NF54 (*black*; n = 4).

Three of 5 volunteers infected with NF135.C10 and 4 of 5 infected with NF54 parasites had a positive thick smear during follow-up. The remaining 3 smear-negative volunteers were qPCR negative for *P. falciparum* for 21 days. In *P. falciparum–*positive volunteers, kinetics of parasitemia for both strains were comparable to those in historical controls (n = 48; Figure 1*A* [9]). Patent parasitemia for NF135.C10 occurred slightly earlier than for NF54, as measured by both thick smear (median [range], 7.0 [7.0–9.0] vs 10.6 [10.6–11] days after infection; *P* = .05, Mann–Whitney *U* test) and qPCR (median [range], 7.0 [6.3–7.0] vs 7.3 [7.0–7.3] days after infection; *P* = .1, Mann–Whitney *U* test). In addition, the peak of the first cycle seemed higher in infected volunteers for NF135.C10 (geometric mean [GM], 1.2 [95% confidence interval {CI}, .61–2.4] parasites/µL) than for NF54 (GM [95% CI], 0.16 [055–.46] parasites/µL; *P* = .06, Mann–Whitney *U* test). In the same 2 groups of volunteers, the GMs (95% CIs) for peak parasitemia were 11 (1.8–73) and 30 (7.7–120) parasites/µL, respectively (*P* = .4). All parasites were cleared from the blood of all volunteers during follow-up, with slopes that were similar for both strains (Figure 1*B*). The PCR identities of both strains were confirmed by culture of smear-positive samples from several randomly selected infected volunteers.

All volunteers, including the smear-negative volunteers, reported solicited adverse events that were considered possibly or probably related to the trial procedures (Table 2), particularly headache, fatigue, myalgia, and nausea, without apparent differences between the 2 groups. One volunteer infected with NF54 reported severe malaise, headache, and vomiting. One infected volunteer in the NF135.C10 group had a decreased platelet count of 146 × 10^9^/L at day 3 after treatment (cutoff, 150 × 10^9^/L), which returned to normal values at routine examination on day 28. Levels of D-dimers did not increase in any of the volunteers before thick smear positivity. Highly sensitive troponin T values were always <0.05 µg/L.

**Table 2.** Adverse Events in Volunteers

	NF135.C10 (n = 3)		NF54 (n = 4)		Smear and PCR Negative (n = 3)	
Adverse Event	Events, No.	Duration, Mean (SD), d	Events, No.	Duration, Mean (SD), d	Events, No.	Duration, Mean (SD), d
Abdominal pain	2	0 (0.0)	0	…	0	…
Arthralgia	0	…	0	…	0	…
Chills	0	…	0	…	0	…
Fatigue	3	2.4 (1.9)	1	3.0 (…)	2	13.5 (9.3)
Fever	1	0.2 (…)	2	0.7 (0.8)	0	
Headache	3	1.5 (2.4)	4	2.3 (2.0)	3	4.6 (3.8)
Itching	0	…	4	3.1 (1.5)	2	5.2 (0.3)
Malaise	0	…	4	2.5 (3.3)	0	…
Myalgia	1	2.7 (…)	3	1.3 (1.4)	2	2.7 (2.4)
Nausea	1	0.1 (…)	3	2 (2.0)	1	0.0 (…)
Vomiting	0	…	1	0.4 (…)	0	…
Any	3	1.3 (1.7)	4	2.2 (1.9)	3	5.7 (5.8)
Grade 3 adverse event						
Headache	0	…	1	4.6 (…)	0	…
Malaise	0	…	1	0.4 (…)	0	…
Vomiting	0	…	1	0.4 (…)	0	…
Any	0	…	1	1.8 (2.4)	0	…

Reported solicited adverse events, collected throughout the postinoculation period, that were considered possibly, probably, or definitely related to the trial procedures.

Abbreviations: SD, standard deviation; PCR, polymerase chain reaction.

T lymphocytes of volunteers successfully infected with either NF135.C10 or NF54 showed similarly increased IFN-γ, tumor necrosis factor, and interleukin 2 recall responses 35 days after infection and the same kinetics for both homologous and heterologous stimulation (Supplementary Figure 2*A–I*). IFN-γ–producing cells were found in both the innate compartment (γδ-T, natural killer, natural killer–T) and the adaptive compartment (CD4 and CD8) with an effector memory phenotype which was generally consistent over time and in both groups (Supplementary Figure 2*J* and 2*K* and data not shown).

## DISCUSSION

We identified and characterized NF135.C10 as the first *P. falciparum* clone of Asian origin for successful infection of malaria-naive human volunteers by CHMI. Clone NF135.C10 consistently produced gametocytes in culture and was able to generate infections in laboratory-reared mosquitoes with high yields of sporozoites. NF135.C10 parasites were clearly distinct from NF54 parasites by genetic marker profiles and were sensitive to the most commonly used antimalarials. Clinical presentation after CHMI and characteristics of *P. falciparum* RBC–specific recall (T-)lymphocyte responses in vitro were similar to those in NF54.

For manufacturing purposes, cultures should ideally produce gametocytes that consistently infect ≥75% of the mosquitoes with ≥10 oocysts, resulting in 10 000–30 000 sporozoites per mosquito. Selection, identification, and cloning of *P. falciparum* field strains that meet those criteria pose technical difficulties. Only after extensive efforts on >70 strains were we able to identify a parasite clone, NF135.C10, that met these criteria and which is geographically and molecularly distinct from NF54. We consider NF135.C10 closely related to its original field strain because of the limited restarts of the culture. We showed that clinical signs and symptoms after infection with NF135.C10 or NF54 were similar despite a shorter prepatent period in NF135.C10-infected volunteers. The observed difference in the prepatent period may represent a true difference in infectivity or may be due to coincidental distribution within the previously observed variation related to the limited number of volunteers.

Notably, not all volunteers exposed to NF54 infected mosquitoes became parasitemic, in contrast to findings in 22 previous CHMI trials infecting 128 naive volunteers with NF54 parasites [10]. Unsuccessful infection after bites from 5 mosquitoes, although rare, has been described elsewhere for 3D7 [11, 12]. Although the exact reason for this low infectivity is unclear, it might be due to a technical disturbance in our cultures leading to unusually low NF135.C10 and NF54 oocyst and sporozoite counts in this particular trial, although the relation of these parameters to infectivity has never been formally established [13]. Surprisingly, all 3 unsuccessfully infected volunteers reported adverse events that were considered possibly or probably related to the trial procedures, which might have been the result of overreporting in an intense follow-up schedule. More studies are required to determine whether 100% infection rates can be achieved and to fully establish NF135.C10 as a heterologous field clone to complement the current CHMI portfolio of *P. falciparum* parasites. We have established master and working cell banks required to produce aseptic, purified, cryopreserved *P. falciparum* sporozoites using NF135.C10 parasites (B. K. L. S. et al, unpublished data), enabling the potential future needle and syringe inoculation of a stable number of sporozoites, in analogy to NF54 [14].

We found similar kinetics and composition of IFN-γ recall responses with homologous and heterologous *Pf*54 and *Pf*135.C10 restimulation, possibly suggesting a role for specific (conserved) antigens in the induction and maintenance of heterologous memory responses against *P. falciparum* [15, 16]. Whether these cross-strain T-lymphocyte responses also translate into or represent cross-strain protective immunity in vivo remains to be investigated.

In conclusion, increasing the portfolio of new *P. falciparum* parasite strains, as achieved here for NF135.C10, will accelerate the evaluation of malaria vaccines candidates by facilitating the downstream selection process for further clinical vaccine development. Moreover, heterologous parasite clones may be a component of whole sporozoite combination vaccines in order to enhance cross-strain protection. Although more trials will be necessary to fine-tune the heterologous CHMI model with clone NF135.C10, the current results will boost the continued application of CHMIs as a crucial tool for malaria vaccine development.

## Supplementary Data

Supplementary materials are available at *The Journal of Infectious Diseases* online (http://jid.oxfordjournals.org/). Supplementary materials consist of data provided by the author that are published to benefit the reader. The posted materials are not copyedited. The contents of all supplementary data are the sole responsibility of the authors. Questions or messages regarding errors should be addressed to the author.

Supplementary Data
