# Supplementary material for: NF135.C10: A New Plasmodium falciparum Clone for Controlled Human Malaria Infections
Source: J Infect Dis. 2012 Nov 27;207(4):656–60. doi: 10.1093/infdis/jis725 (PMC3549599; doi:10.1093/infdis/jis725)
Supplement: Supplementary Data [file supp_207_4_656__index.html]

NF135.C10: a new Plasmodium falciparum clone for controlled human malaria infections — NF135.C10: A New Plasmodium falciparum Clone for Controlled Human Malaria Infections — NF135.C10: A New Plasmodium falciparum Clone for Controlled Human Malaria Infections — Supplementary Data 

# NF135.C10: A New *Plasmodium falciparum* Clone for Controlled Human Malaria Infections

## Supplementary Data

Supplementary Data

**Files in this Data Supplement:**

- Supplementary Data - Docx file
