# Supplementary material for: NF135.C10: A New Plasmodium falciparum Clone for Controlled Human Malaria Infections
Source: J Infect Dis. 2012 Nov 27;207(4):656–60. doi: 10.1093/infdis/jis725 (PMC3549599; doi:10.1093/infdis/jis725)
Supplement: Supplementary Data [file supp_jis725_jis725supp.docx]

**NF135.C10: a new *Plasmodium falciparum* clone for controlled human malaria infections**

Anne C. Teirlinck^1^*, Meta Roestenberg^1^*^±^, Marga van de Vegte-Bolmer^1^, Anja Scholzen^1^, Moniek J.L. Heinrichs^1^, Rianne Siebelink-Stoter^1^, Wouter Graumans^1^, Geert-Jan van Gemert^1^, Karina Teelen^1^, Martijn W. Vos^1^, Krystelle Nganou-Makamdop^1^, Steffen Borrmann^2±^, Yolanda P.A. Rozier^3^, Marianne A.A. Erkens^3^, Adrian J.F. Luty^1±^, Cornelus C. Hermsen^1^, B. Kim Lee Sim^6^, Lisette van Lieshout^3,4^, Stephen L. Hoffman^6^, Leo G. Visser^5^, Robert W. Sauerwein^1^

**Supplementary data**

Contents:

- Supplementary Figure 1 p2
- Supplementary Figure 2 p3
- Supplementary methods p5

1. Characterisation of NF54 and NF135.C10:
2. In vitro immunological assays

**Supplementary Figure 1**

**
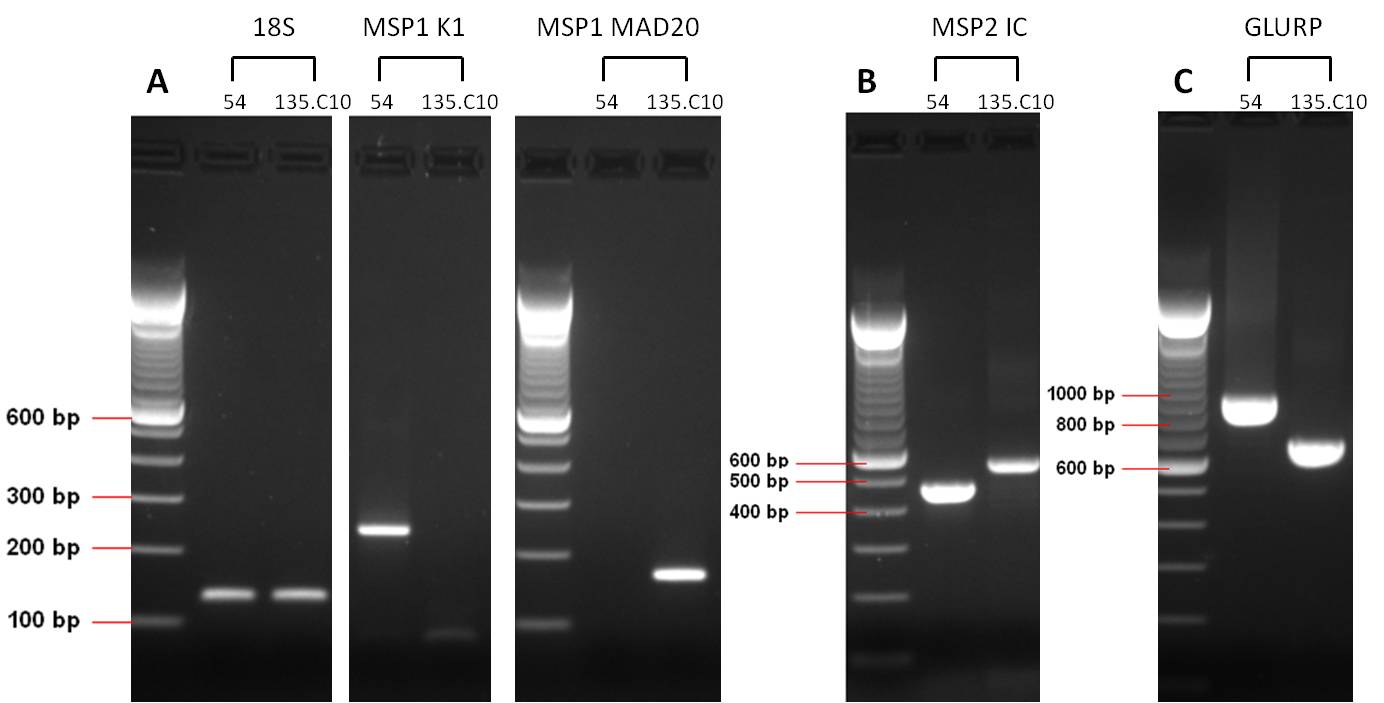
**

**
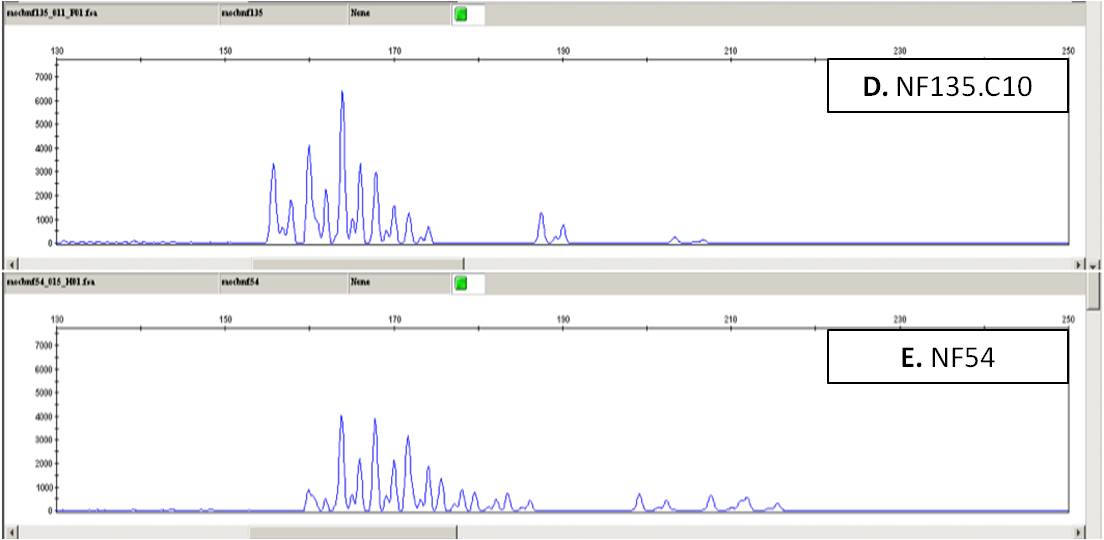
**

**Supplementary Figure 1. Genetic characterization of *Plasmodium falciparum* parasite isolates.**

PCR was performed to assess allelic variation on (**A**) MSP1 K1 and MSP1 MAD20, (**B**) MSP2 IC and (**C**) GLURP for the *Pf* strains NF135.C10 and NF54. Rif repetitive element (*Pf*RR) were characterized by the microsatellite identity test on the genomic DNA of the *Pf* parasite strains (**D**) NF135.C10 and (**E**) NF54. The spectral image generated in the Gene Mapper showing a strain-specific microsatellite fingerprint comprising a unique peak pattern.

**Supplementary Figure 2**

**
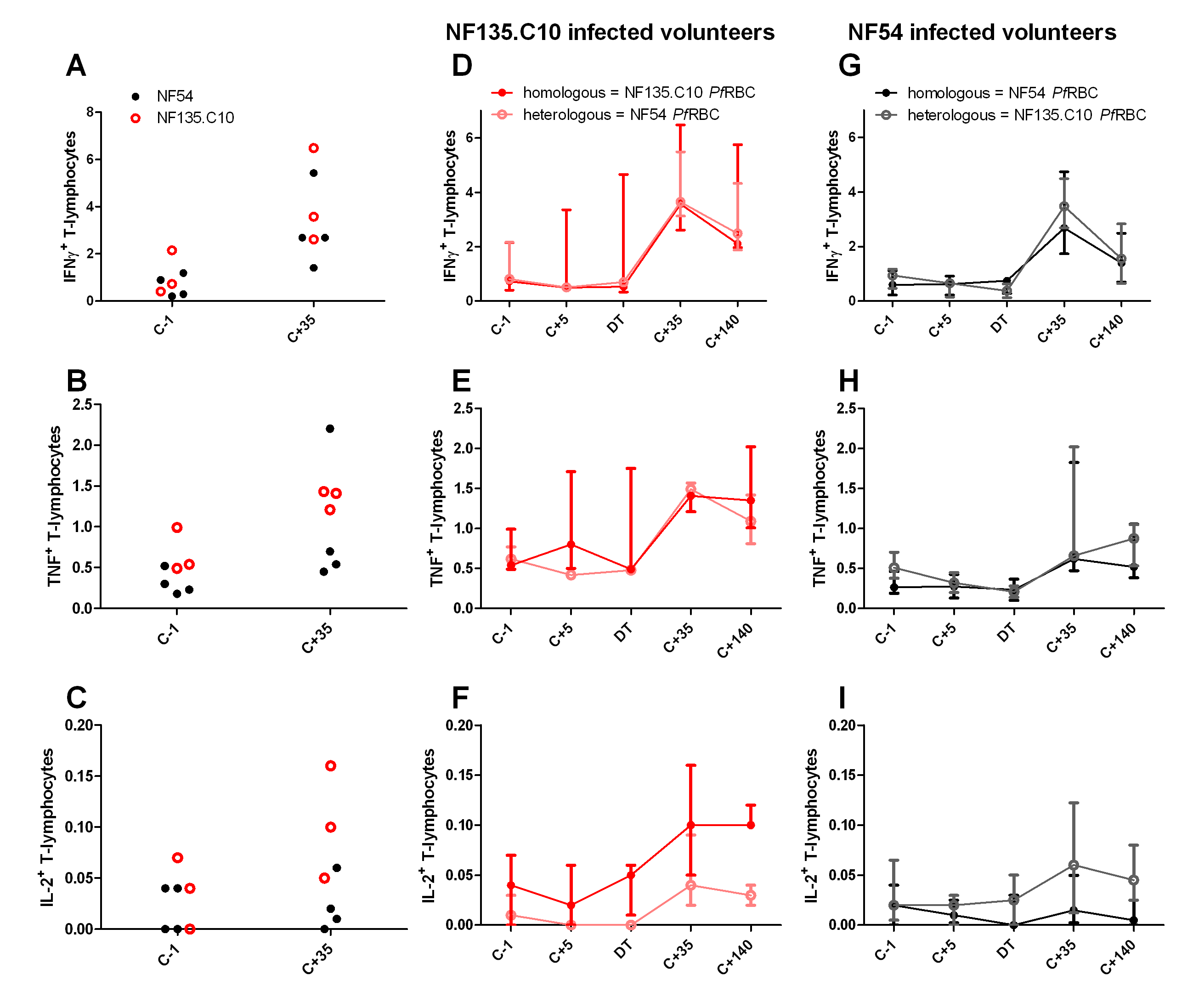
**

**
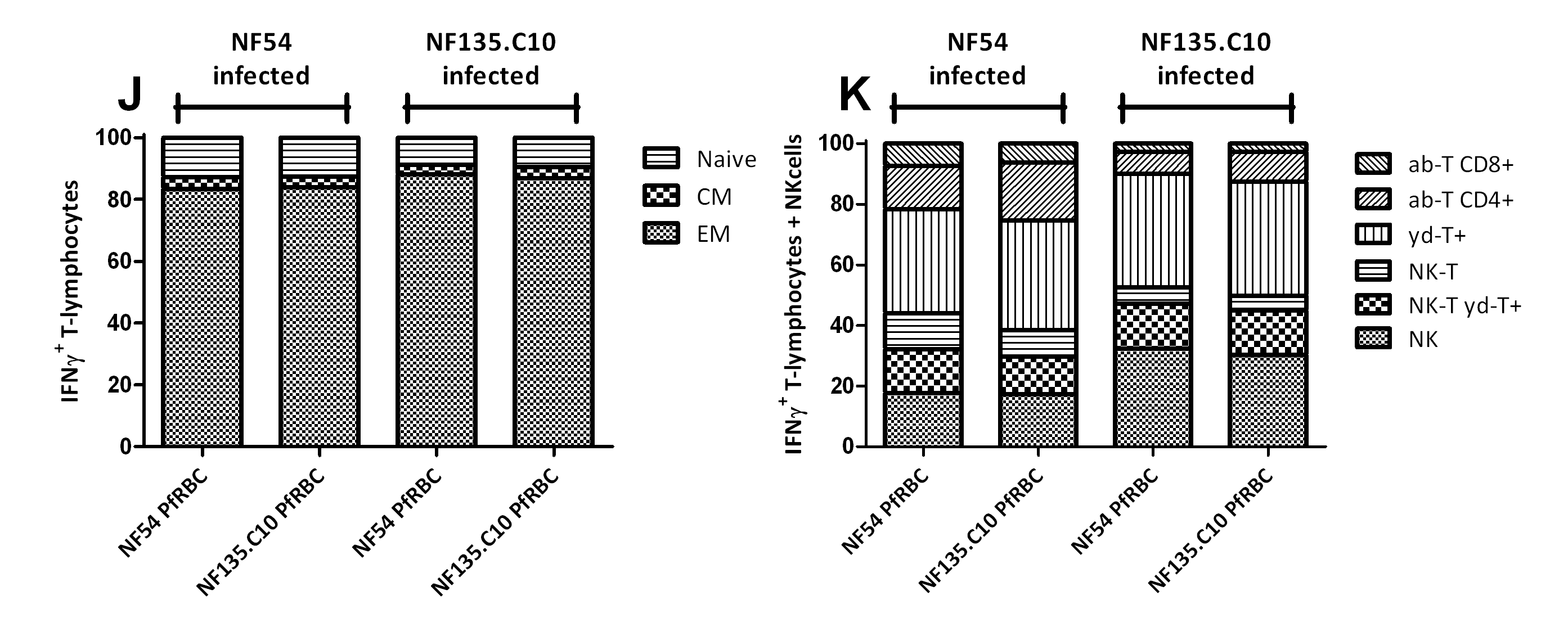
**

**
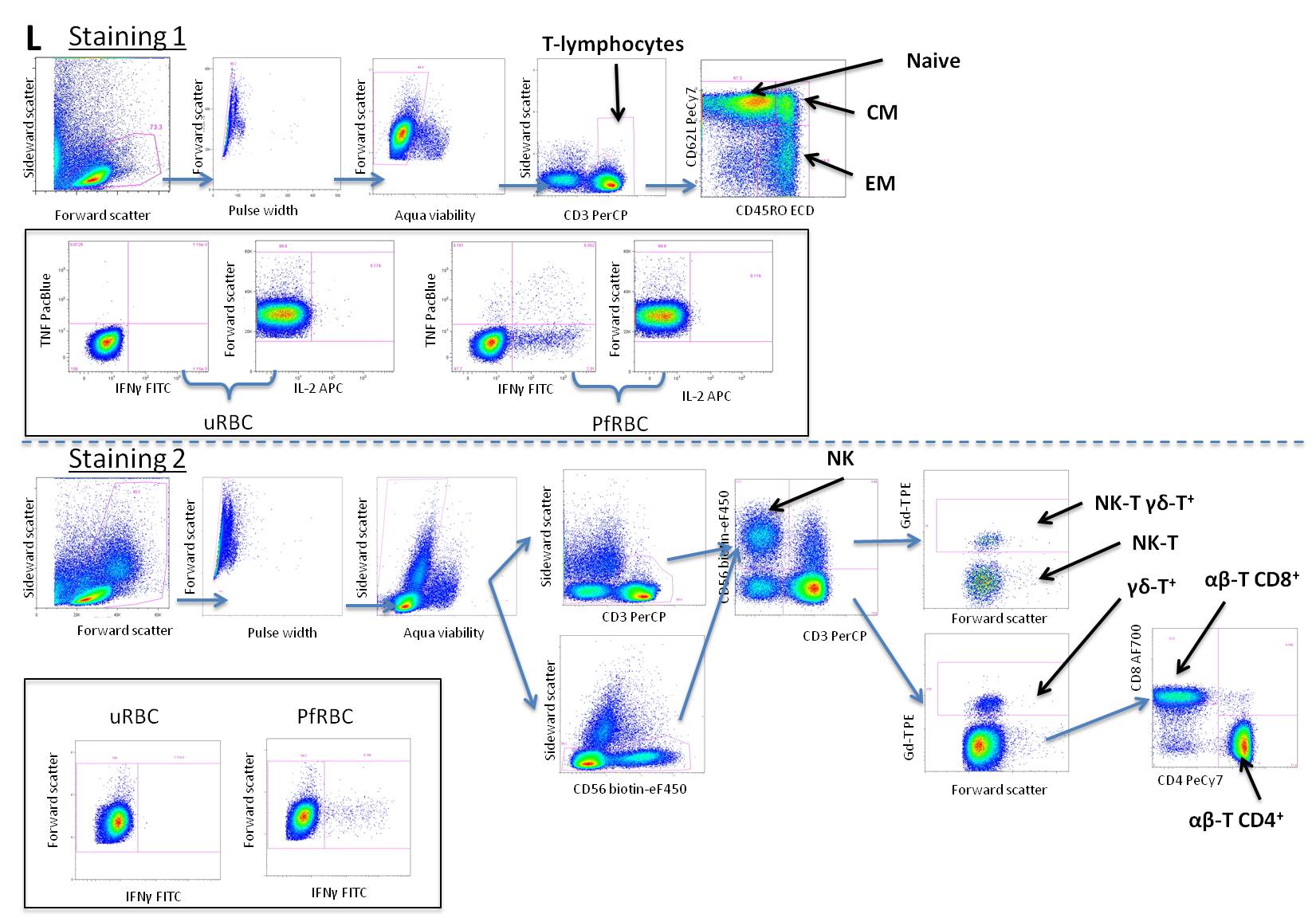
**

**Supplementary Figure 2. Production of IFNγ, TNF and IL-2 by CD3+ T-lymphocytes after in vitro re-stimulation with NF135.C10 or NF54 determined before and after challenge of volunteers.** Time points displayed are: before challenge (C-1), five days after challenge (C+5), on day of treatment (DT) and 35 and 140 days after challenge. PBMCs were stimulated for 24-hour with asexual stage parasites (*Pf*RBC) of NF135.C10 or NF54 or uninfected red blood cells (uRBC). Responses to uRBC were subtracted from responses to PfRBC for every donor on every timepoint separately. Numbers of (**A,D,G**) IFNγ (**B,E,H**) TNF and (**C,F,I**) IL-2 producing cells are depicted as percentages of total T-lymphocytes. **(A-C)** Production of cytokines after homologous stimulation of cells taken before and after challenge. Cells of volunteers infected with NF135.C10 (open red circles) were stimulated with NF135.C10 *Pf*RBC. Cells of volunteers infected with NF54 (closed black circles) were stimulated with NF54 *Pf*RBC. (**D-F**) Production of cytokines after homologous (NF135.C10, closed red circles) or heterologous (NF54, open pink circles) stimulation of PBMCs of volunteers before and after challenge with NF135.C10. (**G-I**) Production of cytokines after homologous (NF54, closed black circles) or heterologous (NF135.C10, open grey circles) stimulation of PBMCs of volunteers before and after challenge with NF54. Symbols indicate individual values from volunteers (A-C) or represent group medians with interquartile range (D-I).

**(J-K)** Mean percentage contribution to the total response are displayed for PBMCs of volunteers successfully infected with NF135.C10 (n=3) or NF54 (n=4) when re-stimulated in vitro with either NF135.C10 or NF54 *Pf*RBC. Columns show mean percentage contribution on day 35 post-challenge of (**J**) effector memory (EM) cells (CD3^+^, CD45RO^+^, CD62L^-^), central memory (CM) cells (CD3^+^, CD45RO^+^, CD62L^+^) and naive T-lymphocytes (CD3^+^, CD45RO^-^)and (**K)** NK cells (CD3^-^ CD56^+^), NK-T γδ-T^+^ cells (CD3^+^ γδ-T^+^ CD56^+^), NK-T cells (CD3^+^ γδ-T^-^ CD56^+^), γδ-T cells (CD3^+^ γδ-T^+^ CD56^-^), αβ-T CD4 cells (CD3^+^ γδ-T^-^ CD4^+^), and αβ-T CD8 cells (CD3^+^ γδ-T^-^ CD8^+^). Responses to uninfected red blood cells are subtracted from responses to *Pf*RBC per contributing cell type.

**(L)** Gating strategy for staining 1 and staining 2 (see supplementary methods). Blue arrows indicate gating strategy, black arrows and names indicate the indentified cell subsets. The black boxes show an example of the gating of cytokines as done for the cell subsets.

**Supplementary Methods**

1. Characterisation of NF54 and NF135.C10:

The polymorphic regions of three *Pf* antigen genes were assessed in a method adapted from Snounou et al. [[1](#_ENREF_1)]. Briefly, parasite DNA was isolated using QIAamp DNA Blood Mini Kit (Qiagen) and amplified with thermoperfect taq polymerase using specific primers for GLURP and the allelic variants MSP1 K1, MSP1 MAD20 and MSP2 IC (all primers from Invitrogen).

For microsatellite mapping, the repetitive element *rif* MS (pfRRM), a polymorphic microsatellite marker [[2](#_ENREF_2)] was used to compare NF135.C10 with NF54. The NF135.C10 and NF54 genomic DNA used was derived from respective master cell banks produced in compliance with cGMPs at Sanaria. PCR amplification was performed using fluorescently labelled forward primer 5’-TACGTTACATTATGTTTTA-3’ and reverse primer 5’-ATATGTATTGCGCTTTTA-3’. PCR products generated from each sample were separated by capillary electrophoresis on an Applied Biosystems 3100 Genetic Analyzer. A spectral image was generated with the Genemapper software V4.0. with each individual peak in the spectral image representing a PCR product. The base pair (bp) size of the amplified sequence products gave a pattern (fingerprint) that was unique to each malaria strain [[2](#_ENREF_2), [3](#_ENREF_3)]. Sensitivity of NF135.C10 and NF54 to dihydroartemisinin (DHA; SigmaTau), chloroquine diphosphate salt (Sigma-Aldrich), proguanil (British Pharmacopia), atovaquone (GSK) and lumefantrine (Novartis) was tested by the Malaria SYBR Green I-Based Fluorescence Assay in triplicate experiments [[4](#_ENREF_4)].

1. In vitro immunological assays

For antigen preparation, asynchronous asexual-stage cultures of NF135.C10 and NF54 parasites were harvested at parasitaemias of approximately 5-10% and mature asexual stages were purified by centrifugation on a 27% and 63% Percoll density gradient [[5](#_ENREF_5)] resulting in preparations of 80-90% parasitaemia with >95% schizonts/mature trophozoites. Preparations of parasitized red blood cells (*Pf*RBC) were washed twice in PBS and cryopreserved at 150x10^6^/ml in 15% glycerol/PBS in aliquots for use in individual stimulation assays. Mock-cultured uninfected erythrocytes (uRBC) were obtained similarly and served as controls.

For cellular immunology, venous whole blood was collected into citrated vacutainer CPT cell preparation tubes (Becton and Dickinson) on the day prior to challenge (C-1), on days 5, 35 and 140 after challenge and on the first day of treatment (DT). Peripheral blood mononuclear cells (PBMCs) were isolated by density gradient centrifugation, washed twice in PBS, enumerated, frozen in foetal-calf serum containing 10% dimethylsulfoxide and stored in liquid nitrogen. After thawing, 500.000 PBMCs were cultured in the presence of NF135.C10 or NF54 *Pf*RBC at a 1:2 (PBMC:*Pf*RBC) ratio for 24 hours with addition of Brefeldin A (Sigma-Aldrich) for the last four hours. Cells were harvested and subsequently stained for viability (Live/Dead fixable dead cell stain kit Aqua, Invitrogen) and surface markers; either **1)** CD4 PE (SK3, BioLegend), CD45RO ECD (UCHL1, Beckman-Coulter), CD3 PerCP (UCHT1, BioLegend), CD62L PeCy7 (DREG-56, eBioscience) and CD8a Alexa Fluor 700 (HIT8A, BioLegend) or **2)** anti-TCR Pan γ/δ-PE (IMMU510, Beckman-Coulter), CD45RO ECD, CD3 PerCP , CD4 PECy7 (RPA-T4, BioLegend), Biotin CD56 (HCD56, BioLegend) and CD8a Alexa Fluor 700. Cells were washed and the second staining panel was incubated with Streptavidin eFluor450 (eBioscience). After washing, all cells were incubated with fixation Medium A (Caltag) and subsequently stained with either **1)** IFNγ FITC (4S.B3, eBioscience), TNF Pacific Blue (MAb11, BioLegend), IL-2 APC (MQ1-17H12, eBioscience)) or **2)** IFNγ FITC, in permeabilization Medium B (Caltag). Cells were read on a CyAn ADP 9-color flow cytometer (Beckman-Coulter) and analysed using FlowJo software (Tree Star, Inc.) version 9.2. For the gating strategy, see **Supplementary Figure 2L**. Gating of cytokine-positive cells was performed based on the Median Fluorescent Intensity (MFI) of cytokine negative PBMCs for each volunteer, time point and stimulus. Responses to uRBC were subtracted from the response to PfRBC for every volunteer on every time point.

1. Snounou G, Zhu X, Siripoon N, et al. Biased distribution of msp1 and msp2 allelic variants in Plasmodium falciparum populations in Thailand. Transactions of the Royal Society of Tropical Medicine and Hygiene **1999**; 93:369-74.

2. Su XZ, Carucci DJ, Wellems TE. Plasmodium falciparum: parasite typing by using a multicopy microsatellite marker, PfRRM. Experimental parasitology **1998**; 89:262-5.

3. Liu S, Mu J, Jiang H, Su XZ. Effects of Plasmodium falciparum mixed infections on in vitro antimalarial drug tests and genotyping. The American journal of tropical medicine and hygiene **2008**; 79:178-84.

4. Johnson JD, Dennull RA, Gerena L, Lopez-Sanchez M, Roncal NE, Waters NC. Assessment and continued validation of the malaria SYBR green I-based fluorescence assay for use in malaria drug screening. Antimicrob Agents Chemother **2007**; 51:1926-33.

5. Rivadeneira EM, Wasserman M, Espinal CT. Separation and concentration of schizonts of Plasmodium falciparum by Percoll gradients. J Protozool **1983**; 30:367-70.
